# Preparation of Efficient Excision Repair Competent Cell-Free Extracts from *C. reinhardtii* Cells

**DOI:** 10.1371/journal.pone.0109160

**Published:** 2014-10-09

**Authors:** Vishalsingh Chaudhari, Vandana Raghavan, Basuthkar J. Rao

**Affiliations:** Department of Biological Sciences, Tata Institute of Fundamental Research, Colaba, Mumbai, India; University of Miami Miller School of Medicine, United States of America

## Abstract

*Chlamydomonas reinhardtii* is a prospective model system for understanding molecular mechanisms associated with DNA repair in plants and algae. To explore this possibility, we have developed an *in vitro* repair system from *C. reinhardtii* cell-free extracts that can efficiently repair UVC damage (Thymine-dimers) in the DNA. We observed that excision repair (ER) synthesis based nucleotide incorporation, specifically in UVC damaged supercoiled (SC) DNA, was followed by ligation of nicks. Photoreactivation efficiently competed out the ER in the presence of light. In addition, repair efficiency in cell-free extracts from ER deficient strains was several fold lower than that of wild-type cell extract. Interestingly, the inhibitor profile of repair DNA polymerase involved in *C. reinhardtii in vitro* ER system was akin to animal rather than plant DNA polymerase. The methodology to prepare repair competent cell-free extracts described in the current study can aid further molecular characterization of ER pathway in *C. reinhardtii*.

## Introduction

Genomic DNA of all organisms is constantly exposed to various endogenous and exogenous damaging agents. Moreover, plants utilize sunlight for photosynthesis and in the process are constantly exposed to the harmful UV irradiation. UV irradiation results in the formation of bulky as well as minor lesions in the DNA. UVC, though much less preponderant than UVB in natural sun light, has been extensively used for efficiently modeling the DNA damage repair responses in cells in laboratory conditions. Bulky lesions are the major kind of UVC induced DNA damage typified by thymine dimers (TDs), which include both the cyclobutane pyrimidine dimers (CPDs) (constituting about 75% of TDs) and less frequent pyrimidine (6–4) pyrimidone dimers (6–4 PPs) [1]–[3]. A very small fraction of minor lesions, that do not distort the double helix are also formed that include thymine glycols (Tg), pyrimidine hydrates, and 8-hydroxo-guanosine (8-oxoG); single- or double-strand breaks, DNA–protein and DNA–DNA cross-links [1], [2]. The TDs are not only mutagenic but also stall DNA replication and transcription, as the DNA and RNA Polymerases cannot read through these lesions [4], [5]. Plants, due to their constant exposure to light and the sessile style of life, have developed a variety of mechanisms to repair such lesions and thereby achieve genomic stability on a continual basis.

Plants are known to utilize two major mechanisms to repair UVC induced damage: photo reactivation repair and excision repair. Photo reactivation repair which is specific for CPDs and 6–4 PPs is a highly efficient and rapid process that employs DNA photolyase to directly reverse TDs by using the energy of light (300–500 nm)[6]. It is a major mechanism of TD repair in plants and several other organisms, but not in mammals. In contrast to photo reactivation repair pathway, the excision repair (ER), a light independent mechanism, recognizes and excises a wide variety of DNA lesions, including TDs [2]. ER is classified into two distinct pathways: (1) **B**ase **E**xcision **R**epair (BER) pathway recognizes lesions that do not distort the DNA double helix, such as the minor lesions generated by UV damage, oxidized bases or damage produced by alkylating agents [1], and (2) **N**ucleotide **E**xcision **R**epair (NER) recognizes lesions that distort the DNA helix and is the major mechanism of TD repair. It is present in most organisms and is highly conserved in eukaryotes. NER is very well characterized in yeast and mammalian systems and has been shown to be orchestrated by about 30 gene products acting in concert to repair a damage containing nucleotide [1]. During ER, the damaged DNA is recognized, excised as a patch of single strand encompassing not only the lesion but also a few normal nucleotides on either side of it, and replaced with new nucleotides via repair DNA synthesis [7], [8]. Therefore, most *in vitro* ER reactions have been reliably assayed by the accompanying incorporation of labeled dNTP precursors specifically in the damaged DNA [9]–[11].

Several UV sensitive mutants have been described in *C. reinhardtii* and *Arabidopsis* [12]–[15], which are defective in ER of UV-damaged DNA. Surprisingly, its molecular characterization has not been accomplished so far although the importance of such studies have been well recognized [13], [16]–[19]. Recently, discovery and functional validation of DNA repair proteins of *C.reinhardtii* was accelerated using insertional mutagenesis approach which identified five proteins including excision repair protein ERCC1 (important for excision step) [20]. Compared to animal counterparts only a few proteins involved in ER have been identified and characterized in photosynthetic organisms. For example, AtRad1 (5′ endonuclease), AtXPD (helicase), AtXRCC1 (x-ray cross-complementing group protein 1) and AtPolλ (repair DNA polymerase) proteins from *Arabidopsis* have been shown to complement the appropriate repair functions both *in vivo* and *in vitro* [21]–[27]. Cell-free extract based repair assays have proved very handy for monitoring ER as evidenced by studies carried out with human and yeast cells, and more recently in *Arabidopsis* [10], [28], [29].

One of the major advantages of using *C. reinhardtii* over higher plants for DNA repair studies has been the ease of isolation of repair deficient mutants [13], [18] and the ease of culturing the cells for biochemical investigations. DNA repair studies in *C. reinhardtii* have also unraveled interesting differences as well as similarities with higher plants and animals [13]. Studies in our laboratory (Ms in preparation) and others [30] have shown that *C. reinhardtii* cells synchronized by growing in a 12 h∶12 h light:dark regime exhibits maximum UV tolerance in cells drawn from the end of dark period and high sensitivity to UVC by the end of 12 h light period, thus uncovering a rhythmic behavior in UVC sensitivity. This could, at least in part, be a result of rhythmic variation in the activity of ER itself.

Reliable *in vitro* assays have been pivotal for studying the molecular details of repair pathways. Seminal studies carried out by Tinland [10], Ariza [31]–[34] and other groups have shown the utility of cell-free extracts in uncovering important molecular details of BER pathway in *Arabidopsis* system. BER in *Arabidopsis* chloroplast was also discovered and studied more recently using similar approaches [35]. Addition of a similar tool-kit to the existing armory of *C. reinhardtii* consisting of a wealth of repair mutants will significantly accelerate molecular dissection of ER pathway. The current study describes the first attempt of demonstrating cell-free extract preparation from *C. reinhardtii* that is proficient in repairing UV induced TDs in plasmid DNA substrates.

## Materials and Methods

### Reagents

Protease inhibitor tablet, creatine phosphokinase, phosphocreatine and casein were obtained from Sigma-Aldrich. Hybond N+, dNTPs and ddNTP were procured from Amersham Pharmacia. Plasmids were purified using Qiagen plasmid purification kit. BCA protein estimation kit was from Thermo Scientific. Bio-11-dUTP was obtained from Jena Biosciences. BrightStar BioDetect Kit for biotin detection was procured from RNA Ambion.

### Cell culture and growth conditions

*C. reinhardtii* cell strains CC-3395 (cwd, arg7–8), CC-125 (wt), CC-888 (uvs1), CC-4055 (rex1, cw15) were obtained from the *Chlamydomonas* Culture Centre (Duke University, NC). Cells were maintained under constant light on Tris-Acetate-Phosphate (TAP) Agar plates at 25°C. For all experiments, cells were inoculated from the plates into 400 ml of TAP in 1l Erlenmeyer flasks at 25°C, under continuous light and shaking at 110 rpm till the cell count was approximately 2× 10^6^ cells per ml.

### Immunofluorescence

A standard protocol described previously was followed [36]. Briefly, *C. reinhardtii* cells were adhered on polyethylenimine (PEI) coated slides and fixed with successive chilled methanol followed by acetone treatment. Cells were rehydrated and blocked in 5% BSA TBST. Primary antibodies, Anti-D1 (rabbit) and Anti-TD (mouse) (against CPD) were acquired from Agrisera and Kamiya biomedicals, respectively. Secondary antibodies used for the above mentioned primary antibodies were Alexa 594 and Alexa 488 (Invitrogen) respectively. Cells were mounted in Vectashield with DAPI. Imaging was done using Zeiss Confocal microscope LSM 510.

### Purification of *C. reinhardtii* whole cell extract

Active cell-free extract from *C. reinhardtii* cells was prepared by modifying a previously described whole cell extract purification protocol employed for *Arabidopsis* system [11]. *C. reinhardtii* cells were grown to a density of about 2×10^6^ cells/ml and then harvested by centrifugation at 3000 rpm for 5 min at 4°C. Thereafter the cells were maintained and processed all through at 0–4°C. The pellet was resuspended in 1× PBS and re-pelleted at 3000 rcf for 5 min. The supernatant was discarded and the cell-pellet was frozen in liquid N_2_. The frozen material was ground using a mortar and pestle at liquid N_2_-temperature till a fine powder was obtained. The powder was suspended gently and completely in 2–3 volumes (w/v) of ice-cold homogenization buffer [25 mM Hepes-KOH (pH 7.8), 100 mM KCl, 5 mM MgCl_2_, 250 mM Sucrose, 10% Glycerol, 1 mM DTT, 2 mM PMSF and protease inhibitor cocktail]. The homogenate was incubated in ice for 1 h and then centrifuged at 9000 rcf for 15 min. The supernatant (slightly turbid and greenish) was dialyzed overnight (changed thrice using hundred times the sample volume of dialysis buffer [25 mM Hepes-KOH (pH 7.8), 100 mM KCl, 17% Glycerol and 2 mM DTT] each time). Protein concentration was measured by BCA protein estimation kit (typically 10 mg/mL of ∼3 ml extract was recovered from 1 liter of starting culture containing about 2×10^6^ cells/ml), and the extract was frozen in small aliquots in liquid N_2_ and stored at −80°C. All the aliquots used in ER assays were thawed only once and used immediately. In our experience, the aliquots were found active for at least about 6 months without any measurable loss in ER activity.

### Preparation of UVC damaged DNA

Plasmid DNA (pGLGAP or pBR322) was dissolved in Tris-HCl (10 mM, pH 8.0) to a final concentration of 1 µg/µl and was exposed to UVC [as measured by a UVC Intensity Meter (ILT77 Germicidal Radiometer, International Light Technologies)] for various times to get the desired fluence.

### Pre-exposure of *C. reinhardtii* to UVC

*C. reinhardtii* cells were exposed to 500 J/m^2^ UVC dose and then grown for 6 h in dark to facilitate the induction of repair genes. Before preparation of cell-free extract, the cells were exposed to light for 30 min to pre-clear the residual level of TDs in cell-free extract via the contaminating residual DNA (by photo reactivation mechanism) that might interfere with the plasmid repair assay.

### *In vitro* repair synthesis

The repair reaction was performed as described previously [11] with several modifications. UVC damaged or undamaged plasmid DNA (1µg of each for 100µl reaction) was mixed in reaction buffer [50 mM Hepes-KOH (pH 7.8), 40 mM KAc, 8 mM Mg(Ac)_2_, 1 mM DTT, 0.4 mM EDTA, 50 µg/mL creatine phosphokinase, 45 mM phosphocreatine, 6% glycerol, 4.8% polyethylene glycol (PEG-8000), 4 mM ATP, 2 mM PMSF, protease inhibitor cocktail, 50 µM each of dATP, dGTP, dCTP, 45 µM of dTTP, and 5 µM Biotin-11-dUTP] on ice. *C. reinhardtii* cell-free extract (140 µg of total protein in 100 µl reaction) was added and the reaction (total volume of 100 µl) was incubated in the dark at 25°C for specified time-intervals. For comparative analysis of various extracts, same amount of protein concentration was used. The reaction was stopped with 20 mM EDTA, and DNA was extracted in 50 µl volume [10 mM Tris-HCL (pH 8.0), 1 mM EDTA solution] using mini-spin silica membrane columns (Epoch Life Sciences). DNA obtained post purification was linearized with EcoRI (Fermentas) wherever mentioned. In some assays, the incorporation was assessed in uncut supercoiled plasmid itself.

### Southern transfer and detection of Biotin label or TDs

DNA (∼400 ng) eluted from the reaction mixture (cut or uncut) was separated on 0.8% agarose gel containing EtBr or Sybr Green (0.5 µg/ml), imaged using Gel-documentation system (BioRad) and transferred onto a nylon membrane (Hybond N^+^, Amersham Pharmacia) by upward capillary transfer method using alkaline transfer buffer (0.4 N NaOH, 1 M NaCl) for 12–16 h. After the transfer, the blot was incubated in neutralization buffer [0.5 M Tris-Cl (pH 7.2), 1 M NaCl] for 30 min. Chemiluminescence detection was done using BrightStar BioDetect Kit according to manufacturer's protocol (Ambion) for biotin detection or using a standard Western protocol using Anti-TD-antibody for detecting TDs. Quantifications were done using ImageJ.

Repair efficiency is expressed quantitatively by dividing the intensity of Bio-dUTP incorporation (chemiluminescence associated with the band) by the DNA level (ethidium bromide or Sybr Green intensity associated with the band). All the repair experiments were reproduced across at least three independent cell-free extracts. Even though the repair efficiency varied somewhat across several cell-free extracts, the specificity towards UVC damaged DNA always remained high. Representative sets are described with quantification across internal controls performed within a set. We have quantified all the results, where the fold-effects are assessed with respect to internal controls within the same experiment.

## Results and Discussion

### *In vivo* repair of TDs formed by UVC damage in *C. reinhardtii* cells

**Figure 1:**
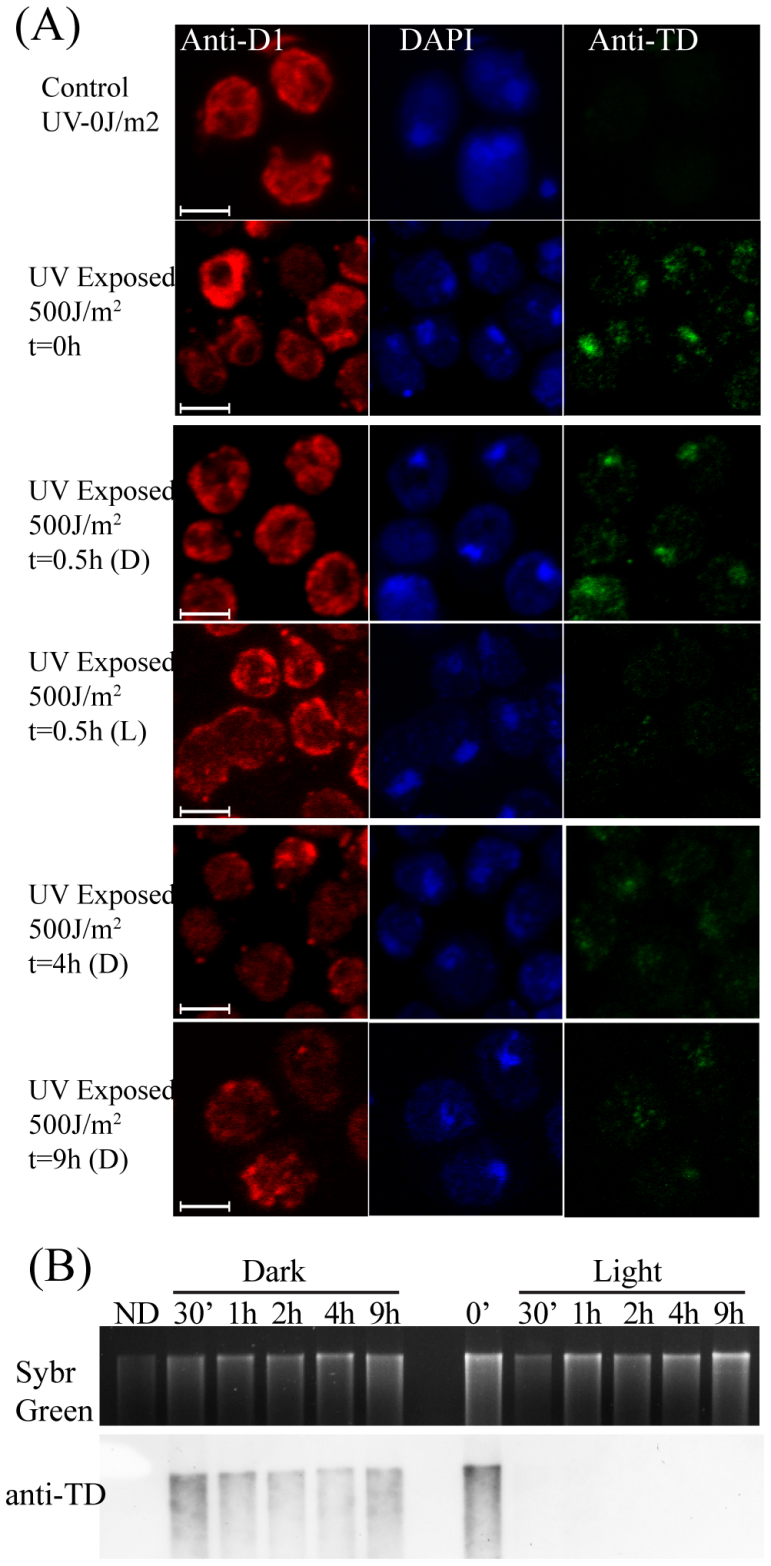
Repair of UVC damaged DNA in *C. reinhardtii in vivo*: Thymine-dimer (TD) depletion assay. *C. reinhardtii* cells were exposed to UVC (500 J/m^2^) and subsequently incubated in dark (D) or light (L) for specified time duration, (Panel A) followed by immunofluorescence using anti-D1-Ab staining (Red for chloroplast), DAPI (Blue for DNA) or anti-TD-Ab (Green, specific to CPDs) staining. (Scale Bar  =  5 µm) (Panel B) Cells were exposed to UVC and incubated in light or dark as described earlier, followed by extraction of genomic DNA, subjected to agarose gel electrophoresis and imaged using Sybr Green (top panel, loading control) (control UVC-untreated cells-No Damage, ND in short). The DNA in the gel was transferred to Nylon membrane, followed by revealing TDs using anti-TD-Ab on the blot.

To assess whether the cellular system in the experimental conditions employed here exhibits robust enough excision of TDs (used synonymously to CPDs here onwards), *in vivo*, we carried out the following experiment. UVC exposure led to high detectable level of TDs in *C. reinhardtii* cells as evidenced by TD-specific antibody detection (Fig. 1a and Fig. 1b). Control sample, not treated with UVC, showed no TD signal. TDs associated with UVC damage were rapidly repaired in *C. reinhardtii* cells following 0.5 h of light exposure, due to efficient photo reactivation mechanism (Fig. 1a and Fig. 1b) [37]. TDs were also repaired *in vivo* during dark incubation (non-photo reactivating) conditions. The signal from anti-TD antibody staining dropped as a function of incubation time in dark, where most of the TDs were removed by ER in about 9 h, at much slower rate than in photo reactivation control (Fig. 1) [37]. This experiment revealed that we are able to score for ER in our experimental conditions, which prompted us to develop a cell-free ER assay system that we describe below. As expected, these results also revealed that photoreactivation kinetics is much faster than ER and that ER efficiency, even though specific to TDs, did not go to completion. The observed *in vivo* efficiency served as a good bench mark for the *in vitro* ER in cell-free extracts prepared from the same batch of cells, as described below.

### Excision repair-synthesis in UVC damaged DNA in *C. reinhardtii* cell-free extracts

We modified the protocol reported by Cordoba-Canero *et al*. used for *Arabidopsis* cell-free extract active in ER against site-specific damage such as uracil or abasic sites [11]. The reported protocol conditions in Cordoba-Canero *et al* paper when implemented directly did not yield ER-active cell-free extracts in our assay conditions. Therefore several modifications were explored, following which some crucial changes yielded consistently robust ER activity from *C. reinhardtii* cells. The centrifugation was done at a lower speed and for shorter time duration (9000 rcf, 15 min) to preserve repair proficient components. Higher speed and time employed in *Arabidopsis* protocol failed to yield repair competent extracts with *C. reinhardtii*, perhaps by depleting the extracts of important components from supernatant. Protein degradation was a significant problem in *C. reinhardtii* extracts when the original protocol of *Arabidopsis* was used. Sufficient amount of protease inhibitors (2 mM PMSF and protease inhibitor cocktail) were mandatory at every step of the extract preparation, including the final assay condition. Instead of linear plasmid, we used non-linearized plasmid DNA (more than 90% of plasmid molecules were supercoiled) as the substrate for all the repair reaction. We used incorporation of Biotinylated dUTPs to measure the repair-synthesis during ER. We also showed concomitant depletion of TDs as a measure of ER. Since the plasmid DNA houses several sites of TDs, depletion of the same can only be correlative to repair incorporation of Bio-dUTPs. ER assays were performed in complete dark to prevent photo reactivation.

**Figure 2:**
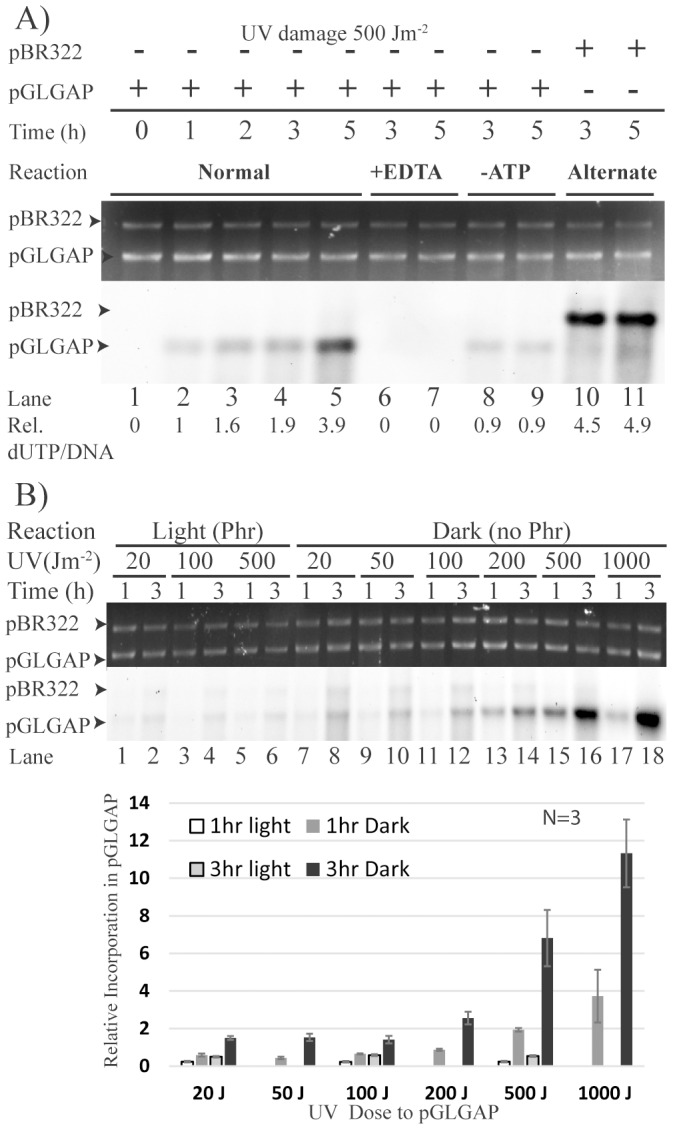
Repair synthesis in UVC damaged plasmid DNA in *C. reinhardtii* cell-free extract. Bio-dUTP incorporation assay: UVC damaged (500 J/m^2^ or as specified) or control undamaged plasmid DNA (as specified) was incubated with cell-free extract of *C. reinhardtii* (CC-3395) for specified time duration. After purification, plasmid DNA was linearized by EcoR1 (Panels A & B) and separated by agarose gel electrophoresis, transferred to a nylon membrane, followed by detection of incorporated Bio-dUTP using ECL assay. Top and bottom panels depict EtBr stained gels (loading controls) and ECL detection blots, respectively. Ratios of Bio-dUTP incorporation signal to its corresponding DNA band intensity in each lane are normalized. Normalized ratios are depicted as values in panel A and as histograms in panel B. A) Negative controls: EDTA (10 mM), -ATP (No ATP and ATP-Regeneration). B) ER as a function of UVC dose and inhibition of ER in light. Plasmid DNA (pGLGAP) was damaged with various doses of UVC (0–1000 J/m^2^ as indicated), followed by standard repair reactions performed in either light or dark incubation for 1 or 3 h (as specified).

UVC damage, marked by the appearance of TDs, was scored by anti-TD-Ab staining in the plasmid DNA. TD signal in the plasmid DNA increased as a function of UVC exposure. Notably, TD signal in the damaged plasmid was proportionately associated with supercoiled form of plasmid DNA (Fig. S1A). Relatively high preponderance of TDs in the supercoiled form and no substantial increase in nicked circular forms with increasing UV dose (Fig. S1B) indicated that no significant nicking of plasmid was caused by UVC doses used. In order to establish specificity of ER directed against UVC damaged DNA, we performed ER time course using a mixture of UVC damaged and undamaged plasmid DNA that can be differentiated by size. DNA was linearized with EcoRI after it was purified from stopped reactions. We observed increasingly higher level of nucleotide incorporation specifically in UVC damaged DNA after 1 to 5 h of incubation (indicated by arrow corresponding to pGLGAP plasmid in lanes 1-5 of Fig. 2A). In the same reaction tubes, UVC untreated control plasmid (pBR322) did not exhibit any detectable repair incorporation suggesting that the assay components were free of nick-translation type of background activity (indicated by top arrow in lanes 2–5 of Fig. 2A). Relative semi-quantitative score of repair efficiency in each lane was calculated by normalising the intensity of incorporated biotin by its corresponding EtBr staining (Rel. dUTP/DNA). These values in a given experiment were then presented in a relative manner normalizing one of the reaction lanes signal to 1. Even though the overall repair efficiency varied somewhat across several preparations of cell-free extracts, the specificity of repair incorporation towards UVC damaged DNA always remained high. Therefore the ER efficiency comparison was more accurate within the experiment. Representative sets are described with quantification across internal controls performed within a set. As expected, repair synthesis was inhibited when divalent cations were chelated by EDTA in the reaction mixture, indicating their requirement in the repair synthesis (Lanes 6–7 in Fig. 2A). ER was also reduced in reaction mixtures that did not contain ATP and ATP regenerating system (CPK and phosphocreatine) (Lanes 8–9 in Fig. 2A). When the UVC damage was switched to pBR322 plasmid substrate from pGLGAP, the repair incorporation was specific to pBR322 (reverse substrate control in lanes 10–11). All these controls put together demonstrated that ER was specific to UVC damaged plasmid and required divalent cations (Mg^2+^ in the current assay conditions) and ATP in the reaction.

### *In vitro* repair is directed towards the removal of thymine dimers

ER synthesis, as expressed by relative Bio-dUTP/DNA ratio in linearized plasmid following assay, increased several fold with increase in UV dose and in time dependent manner (Fig. 2B). A substantial increase (>10 fold) of Bio-d-UTP incorporation (normalized to DNA) was evident in highly damaged DNA containing lanes (pGLGAP in lanes 14, 16 & 18 of Fig. 2B) compared to undamaged controls (pBR322 in the same lanes). Specificity of nucleotide incorporation in ER for TDs was shown by significant drop in Bio-dUTP label when the same reactions were performed in photo reactivating (Phr) conditions (Lanes 1–6 in Fig. 2B). A photolyase catalysed reaction on the same target, namely TDs, being significantly faster, out-competes ER, thereby leading to a decrease in repair incorporation (Compare pGLGAP associated signal in lanes 5 & 6 versus 15 & 16 respectively). Depletion of TDs during ER was scored by comparing the normalized ratios of anti-TD-Ab signal to DNA amounts across the pGLGAP linearized DNA during 0–3 h repair reactions (Fig. S2). A drop in the ratio (from 1.0 to ∼0.5 in Fig. S2) provided a semi-quantitative measure of repair read-out, which indicated that in our conditions of ER about half of TDs were excised (compare ER in lanes 15 & 16 of Fig. 2B with that of drop in TD in Fig. S2), a result that is consistent with the slow rate of TD removal observed *in vivo* earlier (Fig. 1). All these experiments put together reveal that ER reactions under study here are largely specific to dark excision based removal of TDs in the UVC damaged plasmid substrates.

**Figure 3:**
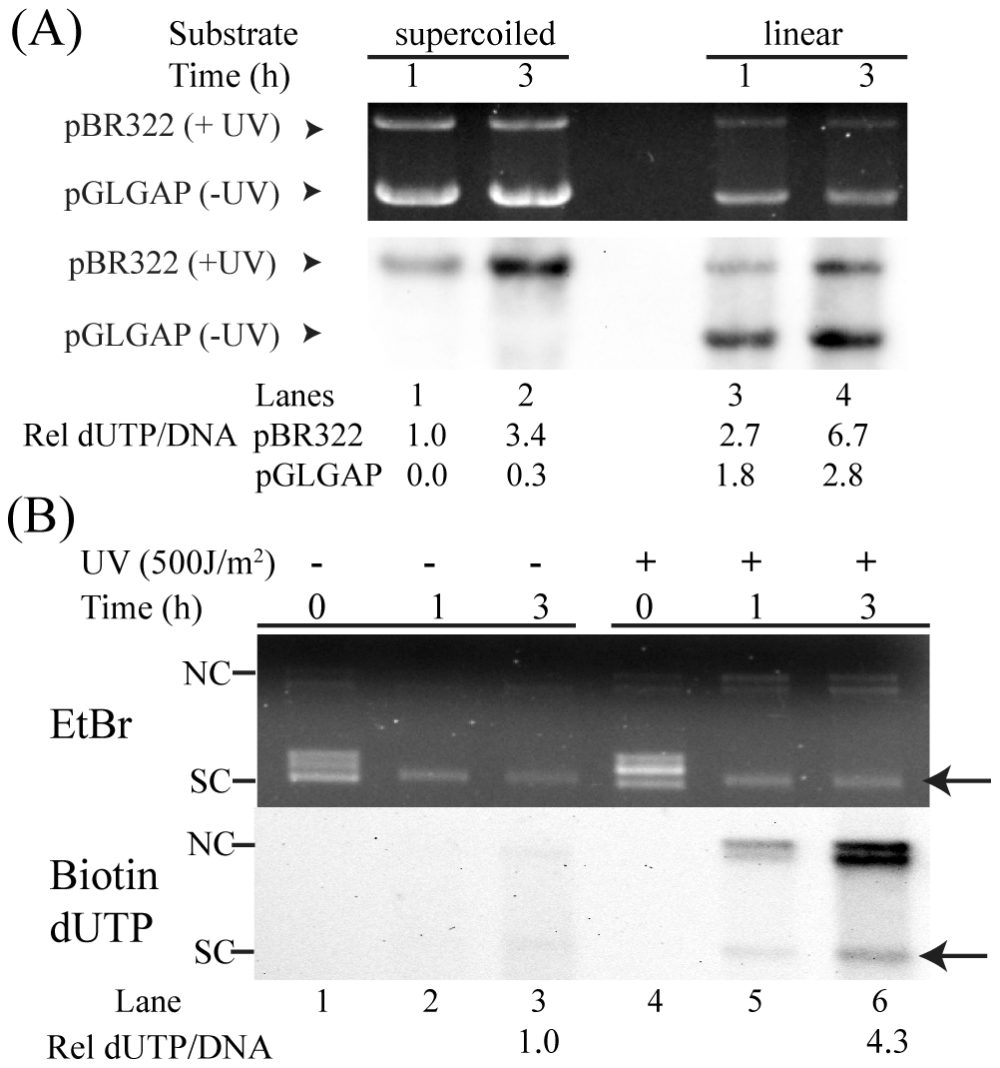
ER labeling in supercoiled versus linear DNA substrates. A) Repair incorporation in linear vs supercoiled plasmid substrate was compared in standard repair reaction. A mixture of plasmids, pBR322 (UV damaged, 500 J/m^2^) and pGLGAP (undamaged control), was used in ER reaction. Supercoiled plasmid was linearized post repair to assess and compare the repair incorporation. B) Repair incorporation in supercoiled DNA. Undamaged or UVC damaged non-linearized plasmid DNA was incubated in dark for specified duration of time for standard repair reaction. Plasmid was not linearized post repair to evaluate repair labelling in different forms of plasmid. Bio-dUTP incorporation efficiency is expressed as a normalized ratio only for supercoiled DNA (marked by arrow). [Nicked Circle (NC); Supercoiled (SC)]. In A & B, top and bottom panels depict EtBr stained gels (loading controls) and ECL detection blots, respectively.

We compared the background labelling in the reactions where the linear as opposed to supercoiled plasmid substrate was incubated in extracts followed by ER assay. It turned out that even undamaged linear plasmid substrate showed high level of incorporation due to end-labelling type of back ground reactions (Lanes 3 & 4 in Fig. 3A). This background signal masks the ER specificity in damaged plasmid. The same mixed plasmid substrates when incubated in supercoiled form showed no such background signal (Lanes 1 & 2 in Fig. 3A). ER was specific to UVC damaged substrate. Although, UVC exposure induced high level of TDs without rendering nicks in the plasmid (Fig. S1), incubation of the supercoiled plasmid in cell extracts generated relaxed forms, revealing DNA nicking activity in the extracts (Lanes 4–6 in Fig. 3B). However, the repair incorporation was always UVC damage specific. To reiterate, background nick-translation related incorporation, as revealed in control undamaged plasmid lanes, was much less than UVC induced repair incorporation (Fig. 2A & B). We could detect biotin labelled supercoiled DNA (ER end product) where ER was analysed without linearizing the DNA reaction products (Indicated by arrow in Fig. 3B). The repair incorporation was specific to UVC damaged DNA containing supercoiled as well as relaxed forms of DNA (Compare lane 3 with lane 6 in Fig. 3B). This result suggested that repair synthesis was followed by ligation of nicks during ER. Mechanistically, this implies that ER is associated with local unwinding at the repair patch which following ligation uncovers as negative supercoiling in the gel assay. Alternately, a negative supercoiling activity present in cell-free extract might generate supercoiled plasmid following ER. The ER activity was however not altered in the cells following UVC exposure. When we compared extracts from UVC treated cells, ER activity was similar to that of control cell extracts (Compare lane 4 with 8 in Fig. S3). It appears that ER itself was not UV inducible in cells.

### Sensitivity of ER system to ddTTP and aphidicolin

**Figure 4:**
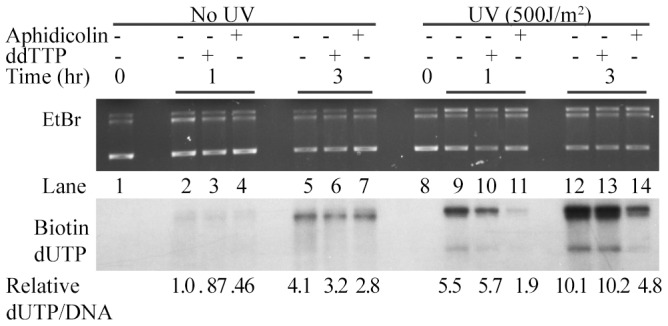
Effect of polymerase inhibitors on ER using *C.reinhardtii* extracts. Standard repair reaction was carried out in the presence of ddTTP (50 µM) or Aphidicolin (200 µM). Only single plasmid (pGLGAP) was used and was not linearized after the ER. UVC dosage, time of incubation and inhibitor conditions are as specified. Ratios of Bio-dUTP incorporation signal (bottom panel) to its corresponding EtBr band intensity (top panel) in each lane are normalized.

Repair synthesis in ER is known to be catalysed by non-canonical DNA polymerases that are not essential for S-phase genome replication. In mammalian cells, the replicative DNA polymerases δ and ε and the translesion polymerase κ are involved in the gap filling step of ER [38]. These polymerases are strongly inhibited by aphidicolin but not by ddNTP [39]. In contrast, ER in *Arabidopsis* was reported to involve the polymerase λ, which was not inhibited by aphidicolin [26]. In fact, it was inhibited by ddTTP, which also inhibits Polymerase β, known to be involved in BER. We tested the ability of aphidicolin and ddNTP to inhibit repair polymerase activity in *C. reinhardtii* ER extracts. Contrary to results observed in *Arabidopsis* system, it was found that although aphidicolin inhibited repair incorporation partially (2–3 fold) (Compare lanes 11 & 14 with 9 & 12 respectively in Fig. 4), ddTTP did not have any effect in the concentrations tested (Compare lanes 10 & 13 with 9 & 12 respectively in Fig. 4). In the lanes 2–7 where background incorporation was seen with undamaged DNA controls, it is hard to assess the inhibitor effects. Plant DNA polymerase α is also known to be aphidicolin sensitive, but ddTTP resistant [39], [40]. Surprisingly, our result suggested that DNA polymerases for ER synthesis in *C. reinhardtii* may be similar to that of mammalian rather than plant systems, which requires further validation by proteomic approaches.

### Cell-free extracts from ER deficient strains exhibit lower repair efficiency than that of wild-type

**Figure 5:**
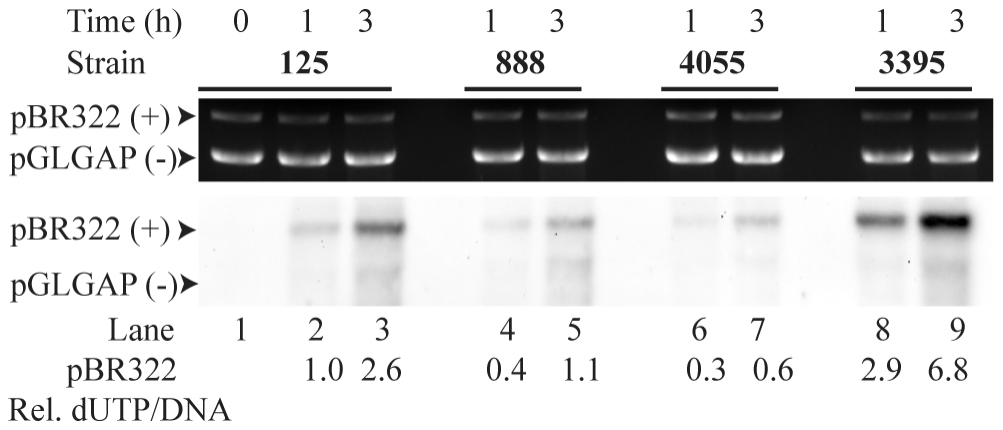
Comparison of ER efficiency in extracts prepared from wild-type (CC-125, CC-3395) and thymine-dimer excision repair deficient strains (CC-888, CC-4055). Standard repair reaction was carried out using extracts (normalized for protein amounts) from CC-125 (WT), CC-888 (uvs1), CC-4055 (cw15, rex1) and CC-3395 (WT) (cwd, arg7-8). A mixture of non-linearized plasmids pBR322 (UV damaged) and pGLGAP (UV undamaged) was incubated for ER. UVC dosage, time of incubation and inhibitor conditions are as specified. Ratios of Bio-dUTP incorporation signal (bottom panel) to its corresponding EtBr band intensity (top panel) in each lane are normalized.

In order to ascertain the utility of ER assay for assessing various uncharacterized repair deficient mutants of *C. reinhardtii*, we tested it in known ER mutant strains. Strains CC-888 & CC-4055 are reported to be thymine dimer excision deficient [41], [42]. However the molecular characterization of the same is still awaited. All the extracts studied so far in this study are from CC-3395, cell-wall deficient strain, from where the preparation of extracts is more effective. However, most of *C. reinhardtii* repair mutants are available in cell-wall positive strains. So we compared CC-888 (Thymine-dimer excision deficient strain) with its wild-type strain CC-125, both being cell-wall positive. ER efficiency of CC-125 was measurably lower than that of CC-3395 at similar protein levels (Fig. 5). It is likely that presence of cell wall affects the quality of extract preparation. However, the difference between wild-type and the mutant (CC-888) was discernible. Mutant extract showed more than 2 fold lower activity than that of wild-type (Fig. 5). We also compared ER efficiency of cell-free extracts from CC-4055 with its wild-type, cell-wall deficient counterpart CC-3395. At similar protein concentration, cc4055 showed about 10 fold lower ER efficiency compared to CC-3395 (Compare lanes 6 & 7 with 8 & 9, respectively in Fig. 5). The residual ER observed in mutants CC-888 and CC-4055 was still specific to damaged DNA (pBR322) suggesting that these strains were either not fully defective in thymine dimer repair or perhaps contained some other UVC damage repair proficiency as well.

In summary, we reiterate the demonstration, for the first time, of excision repair using *C. reinhardtii* cell-free extracts where incorporation of DNA nucleotides ensues specifically in UVC damaged DNA targets. Such repair synthesis is UVC dose dependent and out-competed by photoreactivation steps, thus suggesting that it is largely directed towards TDs. This interpretation was further strengthened by the observation that ER was also associated with concomitant depletion of TDs in UVC damaged plasmid DNA. Importantly, we could detect repaired product as supercoiled form of plasmid, specifically when it was UVC damaged, thus revealing near-completion of excision repair during ER in cell free extracts. Surprisingly, *C. reinhardtii* cells seemed to employ repair DNA polymerase akin to that of animal cells that is inhibited by aphidicolin rather than by ddTTP. Taken together, we show that the cell-free extracts described in the current study are ER competent and can be an excellent system for further molecular characterization of excision repair complexes. A combination of proteomic and genetic approaches that can make an effective use of in *vitro* repair assay in cell-free extracts described in the current study can help delineate the molecular details of excision repair components in *C. reinhardtii* system.

## Supporting Information

Figure S1 **UV damage does not increase nicks substantially in Plasmid DNA.**
(TIF)

Figure S2 **Thymine dimer depletion during ER.**
(TIF)

Figure S3 **No significant induction of repair activity in UV pretreated cell's extract.**
(TIF)
