# Supplementary figures and images for: Preparation of Efficient Excision Repair Competent Cell-Free Extracts from C. reinhardtii Cells
Source: PLoS One. 2014 Oct 9;9(10):e109160. doi: 10.1371/journal.pone.0109160 (PMC4192114; doi:10.1371/journal.pone.0109160)

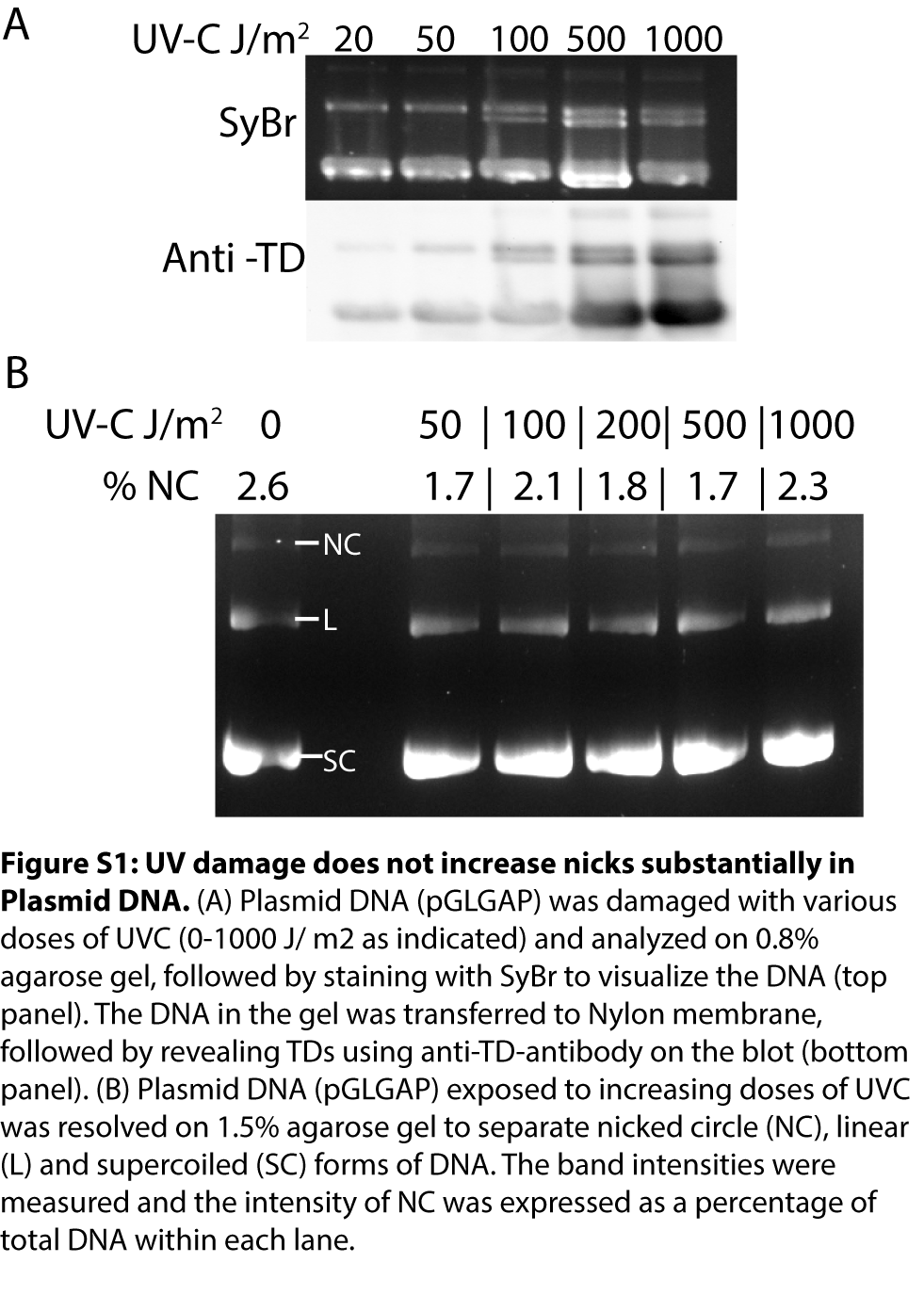

Supplement: Figure S1 — UV damage does not increase nicks substantially in Plasmid DNA. (TIF) [file pone.0109160.s001.tif]

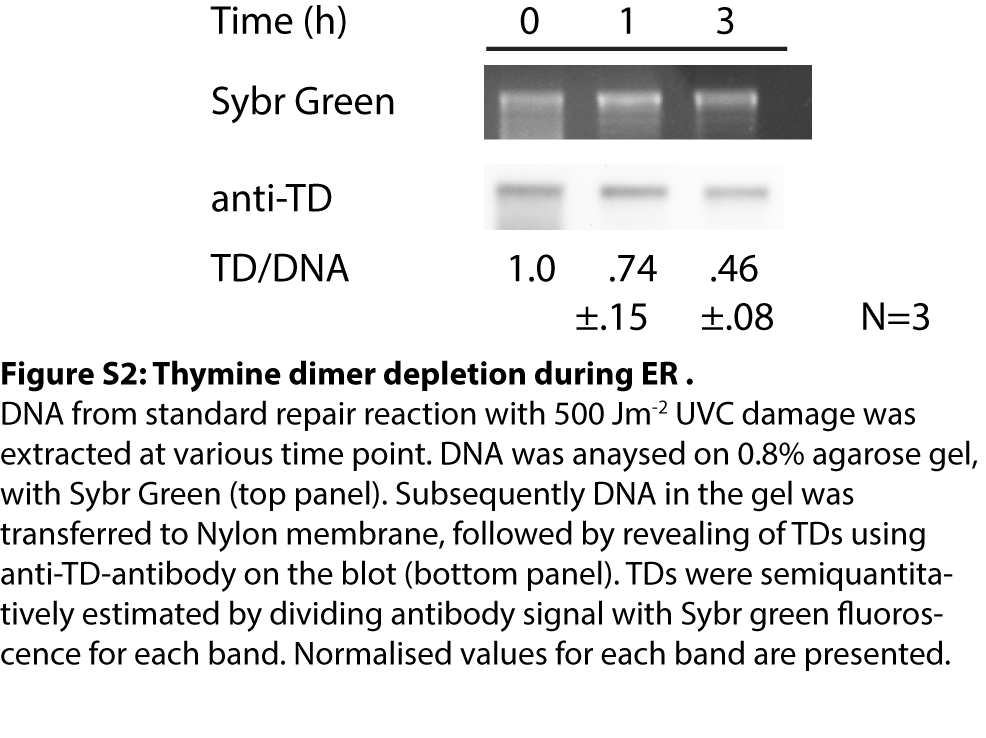

Supplement: Figure S2 — Thymine dimer depletion during ER. (TIF) [file pone.0109160.s002.tif]

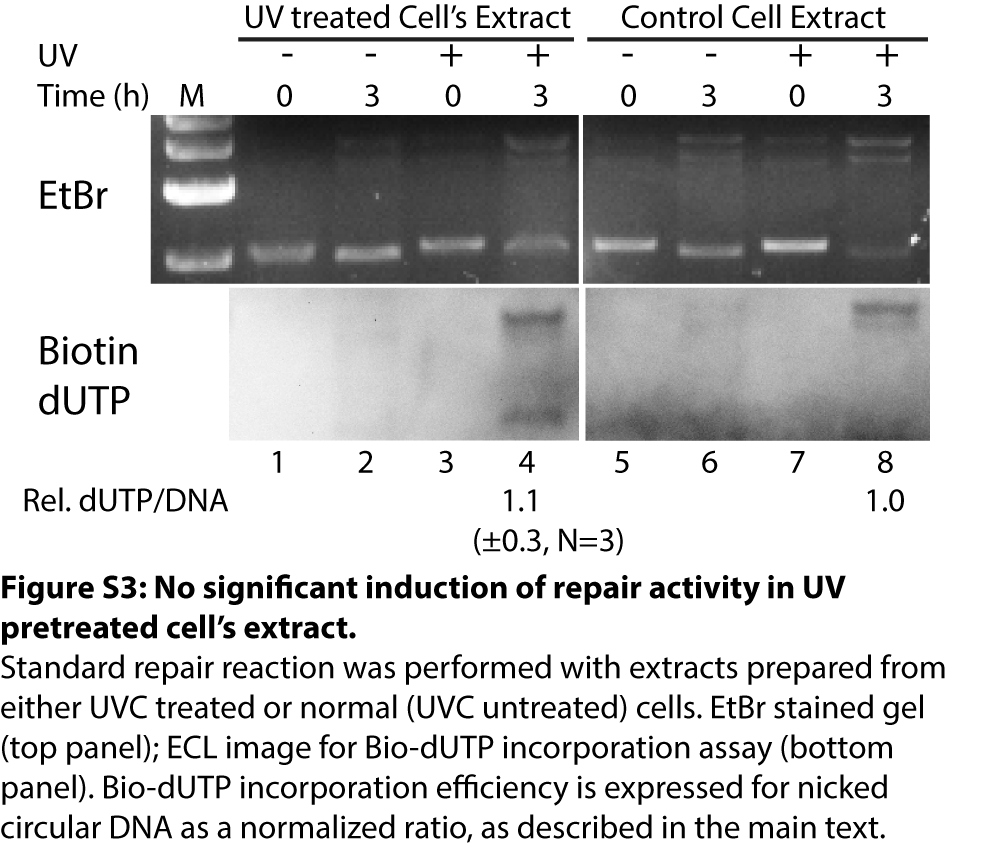

Supplement: Figure S3 — No significant induction of repair activity in UV pretreated cell's extract. (TIF) [file pone.0109160.s003.tif]
